# The role of methanotrophy in the microbial carbon metabolism of temperate lakes

**DOI:** 10.1038/s41467-021-27718-2

**Published:** 2022-01-10

**Authors:** Paula C. J. Reis, Shoji D. Thottathil, Yves T. Prairie

**Affiliations:** 1 Département des Sciences Biologiques, Groupe de Recherche Interuniversitaire en Limnologie, Université du Québec à Montréal, Montréal, QC H2X 1Y4 Canada; 2 Department of Environmental Science, SRM University AP, Amaravati, Andhra Pradesh 522 502 India

**Keywords:** Freshwater ecology, Microbial ecology, Carbon cycle

## Abstract

Previous stable isotope and biomarker evidence has indicated that methanotrophy is an important pathway in the microbial loop of freshwater ecosystems, despite the low cell abundance of methane-oxidizing bacteria (MOB) and the low methane concentrations relative to the more abundant dissolved organic carbon (DOC). However, quantitative estimations of the relative contribution of methanotrophy to the microbial carbon metabolism of lakes are scarce, and the mechanism allowing methanotrophy to be of comparable importance to DOC-consuming heterotrophy remained elusive. Using incubation experiments, microscopy, and multiple water column profiles in six temperate lakes, we show that MOB play a much larger role than their abundances alone suggest because of their larger cell size and higher specific activity. MOB activity is tightly constrained by the local methane:oxygen ratio, with DOC-rich lakes with large hypolimnetic volume fraction showing a higher carbon consumption through methanotrophy than heterotrophy at the whole water column level. Our findings suggest that methanotrophy could be a critical microbial carbon consumption pathway in many temperate lakes, challenging the prevailing view of a DOC-centric microbial metabolism in these ecosystems.

## Abstract (web summary)

Methane metabolism is considered of secondary importance in most lake food webs. Here the authors quantify the contribution of methanotrophs to C cycling in six lakes, finding evidence of a large role of methanotrophs due to their higher specific activity and larger cell size.

## Introduction

Methane-oxidizing bacteria (MOB or methanotrophic bacteria) are well-known for playing a central role in lake methane (CH_4_) budgets and mitigating CH_4_ emissions to the atmosphere^1^. MOB use CH_4_ as source of metabolic energy and structural carbon (C), producing biomass and carbon dioxide (CO_2_) from the oxidation of CH_4_^2^. Since most CH_4_ produced in lakes is oxidized by MOB within these ecosystems^1,3–5^, methanotrophy is a potentially important pathway in the total microbial C metabolism of lakes. Indeed, methanotrophic C consumption is comparable in some cases to the total C use by dissolved organic C (DOC)-consuming heterotrophic prokaryotes (hereafter referred to as heterotrophic prokaryotes, HP) and to the total amount of C fixed by primary production^6–8^. Widespread biomarker and isotopic evidence of CH_4_-derived C in aquatic consumers^9–11^ also point to an important role of methanotrophs in the C transfer to higher trophic levels in lakes. Similarly, CO_2_ produced from CH_4_ during methanotrophy could be an important component of the CO_2_ budget of freshwaters, potentially influencing the balance between CH_4_ and CO_2_ emissions by these ecosystems^12–14^.

Features such as cell abundance, activity, and size affect the contribution of a microbial group to the total C processing at the ecosystem level and are regulated by both intrinsic metabolic characteristics of taxa and environmental conditions^15^. For MOB, the local availability of substrates, namely CH_4_ and oxygen (O_2_), are the major factors regulating their abundance and activity^16,17^. MOB cell abundance in freshwater lakes is generally low (often < 1% of total bacterial cells), but can peak under favorable conditions such as in the oxic-anoxic transition zone of stratified water columns^16,18,19^. Moreover, MOB assemblages have been shown to react to changing environments, blooming quickly under favoring conditions^16,20^. Culture-based laboratory studies and a few field studies suggest that MOB cells can be large^21–23^, which can suggest high metabolic activity and growth rate^24,25^ – as opposed to dormancy and starvation^26^ – as well as susceptibility to preferential grazing.

Although previous studies have pointed to a potentially important role of methanotrophy in lake C budgets and food webs^9^, whole water column quantitative estimations of the relative amount of C that is cycled through methanotrophy under ambient CH_4_ concentrations across lakes are scarce^6,7^. In addition, the mechanism that could enable such importance of methanotrophy in lakes are not obvious given the low MOB cell abundance and CH_4_ concentrations relative to the much more abundant heterotrophic prokaryotes (HP) and DOC^3,27^. To address this conundrum, we combined incubation experiments, microscopy, and sequential water column profiles from six temperate lakes located in the Laurentians region of Québec, Canada. The incubations were performed at ambient but highly variable CH_4_, O_2_, and DOC concentrations to determine MOB and HP metabolic rates, cell abundance and size, and total biomass. For this, we used water sampled from different depths in six oligo- to mesotrophic temperate lakes, which show distinct morphometry and environmental gradients (Supplementary Table 1). We measured rates of aerobic methanotrophy and heterotrophy in parallel incubations, and the methanotrophic and total prokaryotic cells were enumerated and had their size determined microscopically. Further, we used multiple water column profiles of CH_4_, O_2_, and temperature in the studied lakes to determine the importance of methanotrophy relative to heterotrophy at the whole water column level across the summer. To do so, we applied a CH_4_ oxidation kinetics model developed on the same lakes^17^ to the water column profiles and upscaled to the whole water column using bathymetric maps of each system.

We hypothesized that despite their relatively low total abundance, a high metabolic activity of MOB enables an important role of methanotrophy in the C cycling of lakes. We also expected that CH_4_ and O_2_ concentrations exert a strong control on the relative importance of methanotrophy and heterotrophy in lakes. At the microbial scale, we show that MOB cells can outpace heterotrophic metabolism due to a combination of larger cell size and particularly high activity per unit biomass. At the water column scale, we show evidence of a larger role of methanotrophs over heterotrophs in the microbial C metabolism of high-DOC stratified lakes that show a large hypolimnetic volume fraction. Our results indicate that the CH_4_ metabolism may be of higher importance than previously thought in a large suite of the world’s temperate lakes.

## Results and discussion

### Cell abundance, size, and biomass

The cell abundance, cell size, and the total biomass of methane-oxidizing bacteria (MOB) and other prokaryotic cells were determined in the incubations with lake water collected from two or three depths in the six studied lakes. MOB and prokaryotic cells were detected microscopically by catalyzed reporter deposition-fluorescence in situ hybridization (CARD-FISH) and 4′,6-diamidino-2-phenylindole (DAPI) staining, respectively (see Methods section).

**Fig. 1:**
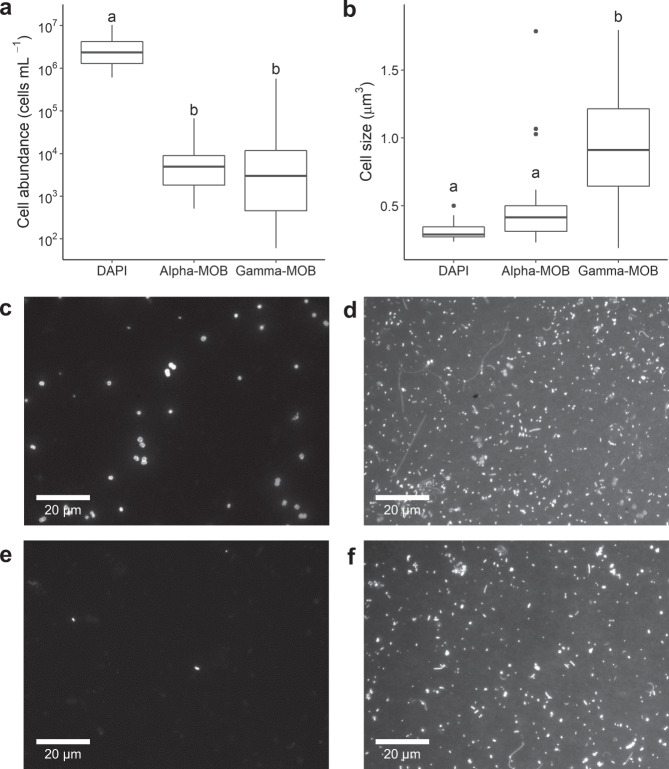
Difference in the cell abundance and size of methanotrophic bacteria (Alpha-MOB and Gamma-MOB) and DAPI-stained prokaryotes across incubations with water from different depths in the six studied lakes. **a** Cell abundance across incubations (DAPI: *n* = 32, Alpha-MOB: *n* = 32, Gamma-MOB: *n* = 32). **b** Average cell size in each incubation (DAPI: *n* = 32, Alpha-MOB: *n* = 32, Gamma-MOB: *n* = 32). Boxplots show median, first and third quartiles (hinges), and 1.5 x interquartile range (whiskers). Different letters indicate significant difference between means in a (*p* < 0.001, ANOVA (F-value = 50.97) and *p* < 0.001, Tukey HSD) and in b (*p* < 0.001, ANOVA (F-value = 35.48) and *p* < 0.001, Tukey HSD). **c**, **d** Microscopic picture of Gamma-MOB cells visualized by CARD-FISH and corresponding picture of the same field of view showing all prokaryotic cells visualized by DAPI staining, respectively. **e**, **f** Microscopic picture of Alpha-MOB cells visualized by CARD-FISH and corresponding picture of the same field of view showing all prokaryotic cells visualized by DAPI staining, respectively. White scale bars in c to f represent 20 μm. Note log scale in *y* axis of **a**.

The average cell abundance of MOB belonging to the Gammaproteobacteria class (Gamma-MOB) and the Alphaproteobacteria class (Alpha-MOB) were orders of magnitude lower than that of other prokaryotic cells (DAPI) (Fig. 1A). MOB cells comprised together on average only 1.3% (min: 0.1, max: 5%) of the total DAPI counts across incubations (Fig. 1A). However, in terms of cell size, the average Gamma-MOB cell (0.9 μm^3^) was three times larger than the average DAPI cell (0.3 μm^3^) (*p* < 0.001; ANOVA and Tukey HSD, Fig. 1B). Alpha-MOB cells (0.5 μm^3^) were also larger than the DAPI cells on average, but the difference was not statistically significant (*p* > 0.05; Fig. 1B). Due to their larger cell size, MOB contributed more to the total microbial biomass than to the total microbial cell counts—Gamma- and Alpha-MOB relative biomasses were on average 3 and 1.5 times higher than their relative cell abundances, respectively. Together, the MOB groups comprised up to 17% of the total microbial biomass across lakes and incubations.

Cell size and biomass are important characteristics that influence the biogeochemical and ecological roles of a microbial group. For instance, microbial groups with larger cells can be more important in terms of C cycling than their abundances alone suggest^28^, and larger bacterial cells are more likely to be grazed and to support food webs than smaller bacterial cells^29^. Indeed, the large average size of Gamma-MOB cells observed here supports previous finding of selective grazing on MOB^22^. Gamma-MOB cells were also significantly larger than Alpha-MOB cells, suggesting a greater importance of the former in reintroducing CH_4_-derived C into food webs, although factors other than size also influence grazing pressure on microbial groups, such as the grazer’s feeding habit or food type^30^. Moreover, the large cell size of Gamma-MOB agrees with the high C consumption and the tight link between CH_4_ oxidation rates and Gamma-MOB abundance across temperate and boreal lakes^27,31^. Yet, the large variability in Gamma-MOB cell size detected here (Fig. 1B) suggests a wide range of cell activity across and within lakes, indicating also a strong environmental control (i.e., substrate concentration, nutrients, etc.) on the methanotrophic metabolism.

### Total C consumption and specific activity of methanotrophy and heterotrophy in incubations

To assess the extent and variation of the rates of C consumption by methanotrophy and heterotrophy across lakes and depths, we performed incubations with water from the epilimnion, metalimnion, and oxic hypolimnion in the four studied lakes showing these well-defined thermal stratification layers, and from the epilimnion and oxic bottom in the other two studied lakes. Methanotrophy was measured through the reduction in CH_4_ concentration over time in unamended lake water, while heterotrophy was determined as the sum of ^3^H-leucine incorporation (production) and heterotrophic O_2_ consumption (respiration) in parallel incubations (see Methods for details).

**Fig. 2:**
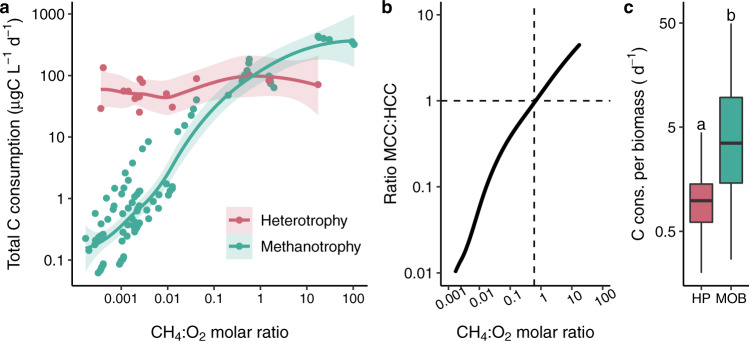
Methanotrophic and heterotrophic carbon (C) consumption rates measured in incubations. **a** Total methanotrophic (*n* = 93) and heterotrophic (*n* = 16) C consumption along the gradient in CH_4_:O_2_ molar ratio. Plotted trend lines are loess curves and shaded area around lines indicates 95% confidence interval. **b** Ratio between total methanotrophic and heterotrophic C consumption (MCC and HCC, respectively)–calculated on the predicted values of the loess curves in a–along the gradient in CH_4_:O_2_ molar ratio. The dashed horizontal line indicates MCC = HCC and the dashed vertical line crosses the *x* axis at 0.6. **c** C consumption per unit biomass (specific activity) of methane-oxidizing bacteria (MOB) (*n* = 31) and heterotrophic prokaryotes (HP) (*n* = 16). Boxplots represent median, first and third quartiles (hinges), and 1.5 x interquartile range (whiskers). Different letters indicate statistical difference (*p* = 0.002, two-sided *t*-test (*t* = −3.3, d.f. = 30.8)). Note log scale of both axes in **a** and **b**, and of *y* axis in **c**.

Methanotrophic C consumption (MCC) ranged between 0.06 and 433 μgC L^−1^ d^−1^ across incubations (Supplementary Fig. 1) and changed monotonically along the gradient of CH_4_:O_2_ molar ratios (Fig. 2A). Heterotrophic C consumption (HCC) was much less variable, ranging between 25 and 134 μgC L^−1^ d^−1^ (Supplementary Fig. 2), and did not change along the gradient of CH_4_:O_2_ molar ratios (Fig. 2A). MCC was up to three orders of magnitude lower than HCC at CH_4_:O_2_ ratios below 0.6 (±0.1) but surpassed HCC at CH_4_:O_2_ molar ratios above this value (Fig. 2B), which is close to the theoretical CH_4_:O_2_ stoichiometry for aerobic CH_4_ oxidation (i.e., 0.5). At high CH_4_:O_2_ molar ratios, MCC was up to six times higher than HCC, and stabilized at the maximum CH_4_:O_2_ molar ratio measured in our incubations (~100) (Fig. 2A). In boreal lakes, others found that CH_4_ oxidation rate also varied as a function of CH_4_:O_2_ molar ratios but the highest rate was observed between ratios of 0.5 and 12, with 12 being the highest ratio measured^7^. By sampling at a wider range of CH_4_ and O_2_ concentrations, we found that methanotrophic rates keep increasing until a CH_4_:O_2_ molar ratio of 100, where they stabilize probably due to O_2_ limitation.

We determined C consumption per unit biomass (specific activity) by dividing the rate of total C consumption by methanotrophy and heterotrophy (i.e., including production and respiration) by the total biomass of methanotrophs and heterotrophs, respectively (see Methods section). The median specific activity of MOB (3.5 d^−1^) was four times higher than that of HP (0.75 d^−1^) across the incubations (Fig. 2C). Assuming a growth efficiency of 30%, our median specific activity rates correspond to growth rates of 1 and 0.2 d^−1^ for methanotrophs and heterotrophs, respectively. These values are within the range of methanotrophic (0.16–5.5 d^−1^)^32–35^ and heterotrophic (0.1–2.1 d^−1^)^36,37^ growth rates reported in the literature. We hypothesize that higher growth rates in MOB could be related to the simpler chemical nature of CH_4_, as opposed to the more complex chemical structure and diverse mixture composing the DOC^38^. While CH_4_ can be readily used by methanotrophs, the degradation of dissolved organic matter depends on its molecular characteristics^39,40^ and may require different steps that involve more than one microbial group^41,42^. This implies that the same amount of C could be consumed faster by methanotrophs than by DOC-consuming heterotrophs at the community level. Even though we measured heterotrophic production through the incorporation of one single labile compound (leucine), heterotrophic respiration rates were measured at in situ DOC concentrations and compositions, thereby incorporating this difference in the chemical complexity between the substrates of MOB and heterotrophs in our experimental approach. However, we acknowledge that our measurements of heterotrophic production rates and specific activity could be underestimated because not all DAPI-stained cells take up leucine in lake waters, a bias inherent to all substrate incorporation methods^43^. In addition, our assessment of MOB specific activity is contingent on the taxonomic coverage of the CARD-FISH probes used. Although the probes used show good coverage of the MOB taxa present in the studied lakes (Supplementary Table 2), MOB specific activity rates could be potentially overestimated if the CARD-FISH probes were unable to detect all the MOB cells present in the samples.

The range of C consumption per biomass (specific activity) was also larger in MOB than in HP (Fig. 2C). This may be due to the larger range in CH_4_ concentration (0.00024–5.46 mg C L^−1^) than in DOC concentration in our incubations (2.6–10.9 mg L^−1^) and in lakes in general. CH_4_ concentrations often vary three to four orders of magnitude with depth within a single lake (Supplementary Table 1), while DOC concentrations are more stable across, and particularly within lakes^44^. As substrate availability is an important regulator of the physiological structure of microbial communities^15^, this natural difference in CH_4_ and DOC concentrations can be a critical factor leading to a naturally higher variability in the level of activity in MOB than in DOC-consuming prokaryotes in lakes. Indeed, we observed a strong response of MOB activity to CH_4_ concentrations, with rates of C consumption per unit biomass increasing along the measured CH_4_ concentration in our incubations (Supplementary Fig. 3). Methanotrophic activity per biomass was limited by CH_4_ until reaching concentrations around 20 μM and started to decrease thereafter possibly due to limiting concentrations of the O_2_ substrate. The highest measured methanotrophic C consumption per unit biomass around 50 d^−1^ at low O_2_ concentrations (Fig. 2C; Supplementary Fig. 3) reveals a surprisingly high specific activity rate, implying that a MOB cell can consume up to 50 times per day the amount of C in its biomass. This suggests a high energy demand of MOB at micro-oxic zones with possible high release of CO_2_ or organic compounds^8^. High excretion of CH_4_-derived organic compounds by MOB has been detected under limiting O_2_ conditions due to the utilization of a fermentation mode^45^. In this case, MOB could sustain diverse microbial populations through the distribution of CH_4_-derived compounds^46,47^ at the micro-oxic zones of the studied lakes, indirectly fueling microbial food webs.

### Relative importance of aerobic methanotrophy at the lake scale

**Fig. 3:**
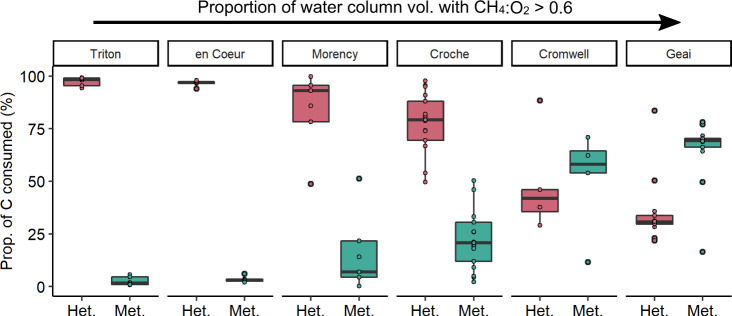
Contribution of heterotrophy (Het., in rose) and methanotrophy (Met., in green) to the whole water column microbial carbon consumption (volume weighted rates) during summer in each studied lake. Each dot represents the fraction of C consumed by heterotrophy or methanotrophy calculated using a profile taken at a different sampling date (Triton: *n* = 6, en Coeur: *n* =  5, Morency: *n* = 6, Croche: *n* = 16, Cromwell: *n* = 4, Geai: *n* = 13). Boxplots show median, first and third quartiles (hinges), and 1.5 x interquartile range (whiskers). Lakes are plotted in order of increasing proportion of water column volume with CH_4_:O_2_ molar ratio > 0.6.

To determine the relative importance of aerobic methanotrophy in the whole water column of the studied lakes, we combined sequential vertical profiles of CH_4_, O_2_, and temperature across the summer with a recent model describing the kinetics of CH_4_ oxidation rate and the bathymetric map of each lake (see Methods section). We found a large variation in the relative contribution of methanotrophy to the whole water column microbial C consumption (i.e., the sum of heterotrophy and methanotrophy) among our study lakes and across months within lakes (0.4–78%; Fig. 3). In two lakes, L. Triton and L. en Coeur, methanotrophy accounted for only a small portion of the total microbial C consumption across the season (up to 5% in L. Triton and 6% in L. en Coeur; Fig. 3). In these same lakes, the fraction of the water column with CH_4_:O_2_ molar ratios > 0.6 was either nil or negligible throughout the summer (Supplementary Fig. 4A, B). In contrast, in the other four lakes, all of which exhibited an important fraction of the water column with CH_4_:O_2_ molar ratios > 0.6 (Supplementary Fig. 4C-F), methanotrophy represented a substantial share of the whole water column C consumption (average: 34%, median: 25%). The highest contribution of methanotrophy was detected in L. Geai, where methanotrophy accounted for up to 78% of the total microbial C consumption (Fig. 3) and where a larger proportion of the water column showed CH_4_:O_2_ molar ratios > 0.6 (Supplementary Fig. 4F). As CH_4_ oxidation rates peak at high CH_4_ and low O_2_ concentrations^17^, methanotrophy was more important in lakes with a larger fraction of the water column volume under these conditions.

A higher variation in the importance of methanotrophy across the summer was observed in the four lakes that thermally stratified and exhibited seasonal hypolimnion, while the importance of methanotrophy was always low in the two lakes that did not form a hypolimnion (i.e., L. Triton and L. en Coeur) (Fig. 3; Supplementary Fig. 4). However, it is important to note that our assessment deals with methanotrophy occurring in the water column and that significant additional aerobic CH_4_ consumption can occur at the sediment-water interface of oxygenated sediments in unstratified lakes^1^ such as L. Triton. In general, the importance of methanotrophy increased along the season with the ongoing water column stratification as the zone with high CH_4_ and low O_2_ concentrations expanded (Supplementary Fig. 4). However, in L. Geai, the lowest relative importance of methanotrophy was detected at the peak of the stratification when the bottom of the lake showed anoxic conditions, limiting methanotrophic activity to a narrower zone of the water column despite high CH_4_ concentrations. Hence, C consumption by methanotrophic bacteria may be limited in the hypolimnion of lakes where the deeper layer is completely anoxic, unless the MOB assemblage has the metabolic capacity to oxidize CH_4_ using electron acceptors other than O_2_^35,48^.

Due to the tight control of CH_4_ and O_2_ concentrations on the role of methanotrophy in the C budget of lakes, it is expected that the importance of methanotrophy varies largely not only along the summer as evaluated here, but also over the annual cycle. In this regard, previous studies have found that methanotrophic C consumption was similar or even more important in the winter than in the ice-free period in small northern lakes^5,6^, and, similarly, that MOB taxa were more abundant in winter than in summer in temperate lakes^49^. Moreover, methanotrophic activity has been reported at the ice-water interface where CH_4_ diffuses from bubbles into surface waters^50^. Other seasonal studies performed during the autumn turnover period have shown that MOB grow rapidly and consume most of the stored CH_4_ as it is released into the oxygenated layer^4,5,20,32^, although the extent of CH_4_ consumption by MOB during autumn may depend on how gradually the water column mixing occurs. In summary, our results and the results from previous seasonal studies point to an important role of methanotrophy in the C budget of lakes over the annual cycle.

### The central role of DOC in regulating the importance of methanotrophy in lakes

**Fig. 4:**
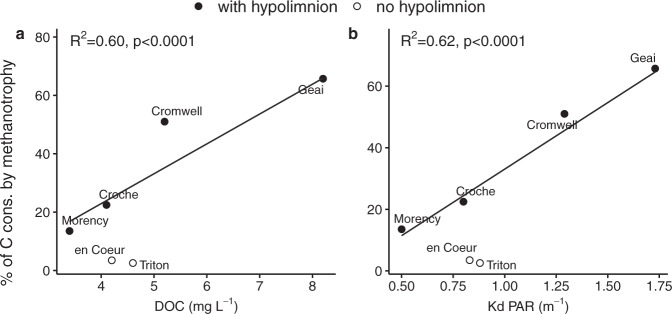
Linear relationships between the fraction of carbon (C) consumed by methanotrophy over summer and lake characteristics in the studied lakes. **a** Relationship with dissolved organic carbon concentration (DOC) (*p* < 0.001, F-test (F-statistic: 55.03, d.f. = 37)). **b** Relationship with the attenuation coefficient of photosynthetically active radiation (K_d_ PAR) (*p* < 0.001, F-test (F-statistic: 60.45, d.f. = 37)). Lines indicate linear relationship considering the lakes with hypolimnion only (i.e., excluding L. Triton and en Coeur). Each dot represents the mean percentage of C consumed by methanotrophy across the summer in each lake.

Among the lakes with seasonal hypolimnion, the average relative importance of methanotrophy during summer increased linearly with the surface dissolved organic carbon (DOC) concentration and with the attenuation coefficient of photosynthetically active radiation (K_d_ PAR) (Fig. 4). These positive relationships suggest that DOC plays a key modulating role in determining the importance of methanotrophy in lakes through its effect on light attenuation. Less light penetration in higher DOC lakes can contribute to shallower thermal stratification and thicker hypolimnion^51,52^ and, as a consequence, higher total CH_4_ stocks and a greater portion of the water column dominated by methanotrophy. In addition, while light may promote methanotrophy in seemingly anoxic waters by allowing photosynthesis and O_2_ production below the oxycline^53,54^, primary production due to deep light penetration may also boost heterotrophic bacterial metabolism^55,56^, not necessarily increasing the relative importance of methanotrophy at the lake scale. Finally, methanotrophic activity has been found to be inhibited by light^3,57,58^, leading to an advantage for methanotrophy in more colored lakes.

The positive relationship between percent of C consumed by methanotrophy and DOC (Fig. 4A) implies that the importance of heterotrophy is inversely proportional to DOC concentrations, which seems paradoxical since DOC is the substrate for heterotrophic microbial metabolism. However, DOC concentration is used here as a proxy for water color since higher DOC lakes are usually also darker due to humic substances (Supplementary Fig. 5), and DOC concentration values are much more often available than DOC quality or water color measurements. Besides its light absorption effects discussed above, colored DOC may be less labile to heterotrophic bacterial degradation^39,59^, having thereby a dual positive effect on the relative importance of methanotrophy to the total microbial C consumption in lakes. Yet, due to the strong dependency on DOC quality and the limited geographical coverage of our study, the relationship between the importance of methanotrophy and DOC concentration unraveled here may not necessarily hold true in other geographical regions where the DOC is not dominated by humic substances or DOC quantity and water color do not correlate.

### Methanotrophy and the microbial C metabolism of temperate lakes

Despite the well-recognized central role of methanotrophy in constraining lake CH_4_ emissions to the atmosphere, our study shows that its relevance to the overall cycling of C at the whole-lake scale was underestimated. Likewise, while several studies indicated a potentially large role of methanotrophy in lake food webs based on biomarkers and stable isotopes, our quantitative confirmation of its importance to the overall lake microbial metabolism demonstrates that despite their lower abundance, MOB cells can outpace heterotrophic metabolism due to a combination of larger cell size and particularly high activity per unit biomass. At the whole water column scale, the importance of methanotrophy during summer depends on the spatial extent of the hypoxic layer with high CH_4_ concentrations (high CH_4_:O_2_ molar ratio), which is in turn influenced by lake stratification and DOC concentration and color. The observed relationship between the relative importance of methanotrophy and DOC suggests that methanotrophy could constitute the main (>50%) microbial C processing pathway in stratified lakes with DOC > ~6.5 mg L^−1^ (Fig. 4A). Considering that the median DOC concentration across lakes around the globe is 5.7 mg L^−1^ ^44^ and that many of these lakes stratify, methanotrophy may be a more significant microbial C processing pathway than previously thought in a large suite of the world’s temperate lakes. These findings challenge the current understanding of a DOC-centric microbial loop^60^ and the exclusive role of DOC respiration in the CO_2_ supersaturation in lakes^37^. Moreover, as lakes get warmer and browner^61–63^, our results suggest that methanotrophy has the potential to become an even more important and widespread microbial pathway. The recognition of the importance of the CH_4_-driven microbial pathway may have not only biogeochemical consequences to lake greenhouse gas emissions but also to the structure and functioning of aquatic food webs.

## Methods

### Study sites and sampling

We sampled six temperate lakes located in the Laurentians region of Québec, Canada during the summer in 2015 and 2016. These lakes largely differ in size, maximum depth, DOC concentration, and other characteristics (Supplementary Table 1). In 2015, water column profiling of temperature, O_2_, and CH_4_ concentrations were performed in the six studied lakes from May to November. Additional profiles were taken in 2016 between June and September in L. Croche and Geai. Temperature and O_2_ measurements were made at each meter using a YSI probe (Yellow Springs Instruments, USA; O_2_ detection limit: 0.2 mg L^−1^) and the concentration of CH_4_ was determined using the headspace technique followed by gas chromatography (GC; 8A/GC-2014, Shimadzu, Japan; CH_4_ detection limit: 0.1 ppm) as described in Thottathil et al.^3^. Vertical profiles of photosynthetically active radiation (PAR) were performed once in each lake in 2015 using an underwater light sensor (LI-192, LI-COR Biosciences, USA). PAR attenuation coefficients (K_d_) were calculated as the absolute linear slope between depth and the natural logarithm of the measured radiation. Incubations for the determination of methanotrophy and heterotrophy C consumptions were performed during the summer in 2016. For this, water was collected with a peristaltic pump from the epilimnion, metalimnion, and oxic hypolimnion in the lakes showing three well-defined layers (Morency, Croche, Cromwell, and Geai) and from the subsurface and bottom in lakes Triton and en Coeur, covering a large gradient in CH_4_ and O_2_ concentrations (0.02–455 μM and 3–265 μM, respectively). The water was pumped into acid-washed collapsible bags until overflow and were kept cold and in the dark for 2 to 4 h until arrival at the laboratory. In the laboratory, water samples were gently transferred to incubation flasks with the aid of a tube (connected to the valve cap of the collapsible bags and inserted into the bottom of the flasks) by positioning the flasks below the bags so that the water flowed into the flasks by gravity.

### Methanotrophic C consumption rate

Since CH_4_ is the sole source of C and energy to obligate methanotrophs, we determined total aerobic methanotrophic C consumption (MCC) by measuring CH_4_ oxidation rates in incubations with untreated lake water. In the laboratory, ten 500-mL flasks equipped with O_2_ optodes (Fibox 3, PreSens, Germany) per sampled depth were filled with lake water allowing to overflow and sealed with silicone stoppers without headspace. Flasks were incubated at in situ temperature (± 2 °C) in dark circulation water baths for at least 1.6 and up to 8 days, and duplicate flasks were sampled three to five times during each incubation for the determination of CH_4_ concentration. CH_4_ concentration was measured by the headspace equilibration technique in 60 mL gas-tight syringes with ultra-high purity zero air (Praxair Canada Inc., Canada) (1:1 water sample and zero air ratio). Syringes were vigorously shaken for 2 min and then the headspace gas was transferred to pre-evacuated vials (Labco Ltd. UK). The CH_4_ partial pressure in the headspace gas was measured through cavity ring-down spectrometry (CRDS – Picarro G2201-i, Picarro Inc, USA) or gas chromatography in the case of samples exceeding 200 μatm of CH_4_ in the headspace gas because of possible interference of laser paths in CRDS at high CH_4_ concentrations. Then, the original CH_4_ partial pressure in the water was calculated using the water sample to zero air ratio, the incubation temperature, and the temperature of the water in the syringe during equilibration using solubility constants^64^. Finally, the dissolved CH_4_ concentration in the water was calculated by multiplying the CH_4_ partial pressure by a temperature-dependent Henry’s law constant^64^.

CH_4_ oxidation rates were determined by multiplying the slope of the first order CH_4_ concentration decay curve (*k*) by the observed CH_4_ concentration at each time point of the experiment. This approach allowed that the rates were attributed to the current MOB community, which was quantified at the initial and final time point of incubations, although microscopic verification showed that MOB abundance did not change substantially during most incubations despite their duration (Supplementary Fig. 6). In addition, the decay curves of CH_4_ concentration over time were near perfect log-linear in incubations (*R*^2^ > 0.92)^17^, implying that different incubation duration would not have yielded different rate constants (*k*). Moreover, the measured CH_4_ oxidation rates represent actual rather than potential rates since the incubations were performed at in situ CH_4_ and O_2_ concentrations with minimal changes in concentration between the sampling and the start of incubations (Supplementary Fig. 7) and in the presence of potential grazers (unfiltered waters). By including grazers in the incubations, MOB cells may have been consumed during the experiments as it can occur in the natural lake settings. Bacterial grazers in the studied lakes include filter-feeding Cladocera (*Daphnia* sp., *Bosmina* sp., *Holopedium* sp.) and rotifers (*Keratella* sp., *Kellicottia* sp., *Polyarthra* sp.)^65^. CH_4_ concentration and carbon stable isotopic signature were significantly (*p* < 0.0001) and strongly (*R*^2^ > 0.86) linearly correlated across incubations, which indicated that decreases in CH_4_ concentration were due to microbial CH_4_ consumption and that CH_4_ production was very unlikely or minimal in the incubations. At the start and end of each incubation, water was collected for the identification and enumeration of MOB and other prokaryotic cells as described below.

### Heterotrophic C consumption rate

Total heterotrophic C consumption (HCC) was determined as the sum of heterotrophic bacterial production (HBP) and respiration (HBR). HBP was measured by incorporation of ^3^H-leucine performed with the same water of the start of incubations for methanotrophic C consumption following standard protocol^66^. In summary, three replicates of 1.5 mL of lake water from each depth and one trichloroacetic acid (TCA)-killed control were inoculated with ^3^H-leucine at a final concentration of 20 nM and incubated for 1 h in the dark at room temperature (21 °C). ^3^H-leucine incubations could not be performed at in situ temperatures due to logistical reasons, potentially overestimating HBP particularly in the meta- and hypolimnia where in situ temperatures are lower. The effect of such possible overestimation of HBP is that our assessment of the relative importance of methanotrophy at the whole water column level is therefore conservative. The leucine concentration used in incubations was chosen according to previous measurements performed in L. Croche and other lakes in Quebec^28^. Incubations were stopped by the addition of TCA and vials were kept at 4 °C for less than one week until processing through the centrifugation method^66^.

HBR was measured by the change in dissolved oxygen (O_2_) concentration in 500 mL-flasks equipped with O_2_ optodes (Fibox 3, PreSens, Germany) filled with the same water of the start of incubations for methanotrophy but filtered through 1.2 µm pore size filters to remove algae, protists, and zooplankton. HBR incubations were held alongside the methanotrophy incubations at in situ temperatures (±2 °C) in dark circulation water baths for at least 48 h and the dissolved O_2_ concentration was measured three to five times. Respiration rates were calculated as the absolute slope of linear regression of O_2_ (mg  L^−1^) vs. time (h). Rates of O_2_ consumption were converted to C consumption using a respiratory quotient (RQ) of 1. CH_4_ concentrations and carbon stable isotopic signature remained stable in HBR incubations, indicating negligible O_2_ consumption by CH_4_ oxidation in the filtered incubations. Because filtration can reduce the microbial biomass in incubations thereby underestimating the total microbial respiration^67^, we calculated the total microbial respiration rate from the measured filtered respiration rate by multiplying the latter by the factor of microbial biomass reduction due to filtration measured in each individual incubation setup. This filtration factor was determined microscopically by the reduction in non-MOB DAPI biomass between the start of unfiltered and filtered incubations in each sampled lake and depth (Supplementary Fig. 8). Filtered DAPI biomass could not be determined for the meta- and hypolimnion of L. Geai due to logistical issues; thus, we applied the same filtration factor measured in the epilimnion to the other layers of this lake.

### Methanotrophic and total prokaryotic cells enumeration, size, and biomass

Methanotrophic and total prokaryotic cells were microscopically quantified at the start and end of each incubation. Methanotrophic bacterial cells were identified by catalyzed reporter deposition-fluorescence in situ hybridization (CARD-FISH) with specific probes^68^ for the detection of aerobic methanotrophs in the Alpha- and Gammaproteobacteria classes (Supplementary Table 2) and total prokaryotic cells were identified using 4′,6-diamidino-2-phenylindole (DAPI) staining. To do so, water samples from the start and end of unfiltered and filtered incubations were fixed with buffered paraformaldehyde (final concentration of 1%) and stored at 4 °C overnight until filtration. Between 2 and 6 mL of each sample (depending on the cell density of each lake and sampled depth) were filtered through 0.2 μm polycarbonate filters (Millipore GTTP, 25 mm), which were kept frozen until analysis. Cells were attached with 0.1% agarose and permeabilized with lysozyme and achromopeptidase. Hybridization was performed at 35 °C overnight with 40% formamide hybridization buffer, and the catalyzed reporter deposition was done with Alexa Fluor 488 labeled tyramide (ThermoFisher Scientific) for 20 min at 46 °C in the dark. DAPI staining was performed during slide preparation by adding DAPI to a glycerol-based mounting medium (4:1, Citifluor:Vectashield) at a final concentration of 1 μg mL^−1^. CARD-FISH hybridized and DAPI stained cells were then visualized at 630x magnification under an automated epifluorescence microscope (Zeiss Axio Imager.Z2m; Carl Zeiss MicroImaging, S.L., Barcelona, Spain) and cell counts were determined using the ACME tool3 software^69^. Cells were counted in 24–55 (average 46) quality-checked and independent fields of view per filter. By applying a minimum area threshold of 22 pixels (=0.22 μm^2^) during cell enumeration, we excluded eukaryotic nuclei and viruses from the DAPI counts. However, DAPI stained cells can also include Archaea^70^ and thus represent the total prokaryotic abundance rather than solely the bacterial abundance. The CARD-FISH probes that we used have been widely applied to detect methanotrophic bacterial cells in lakes^18,19,22,35,53,71^ and show very good coverage of the methanotrophic genera commonly found in these ecosystems as well as in two of the studied lakes (Supplementary Table 2). The detection of non-target (non-methanotrophic) taxa by the CARD-FISH probes was very unlikely since these taxa were not detected at all or detected in extremely low abundance in the studied lakes (Supplementary Table 3). Nevertheless, we acknowledge that measurements of MOB abundance in this study could be potentially biased if some of the studied lakes harbored methanotrophic taxa that are not covered by the CARD-FISH probes used.

Cell size was measured for every cell (DAPI-stained: *n* = 5334802, Alpha-MOB: *n* = 24825, Gamma-MOB: *n* = 15686) in pixels using the ACME tool software^69^ and then converted to µm^2^. Measurements were done in the DAPI subset for MOB (cells with positive signals for CARD-FISH and DAPI, visualized in the DAPI channel) and in the DAPI set for other prokaryotes to avoid bias from different staining methods on the cell size determination. Bacterial volume (µm^3^) of both MOB and other prokaryotes was calculated from the measured average cell size per sample assuming a spherical shape of bacterial cells (measured mean circularity of all cells: 0.71 ± 0.17; circularity of 1 indicates perfect circle). Bacterial biomass was then calculated from cell volume assuming a bacterial C content factor of 63 fgC µm^−3^ ^72^.

### C consumption per biomass (specific activity)

C consumption per unit biomass by methanotrophy and heterotrophy was calculated using the measured rate of total C consumed by methanotrophy (MCC) and heterotrophy (HCC) in incubations and the measured total biomass of MOB and other prokaryotes as described above. For that, we assumed that all non-methanotrophic bacterial cells (cells with positive signal in the DAPI channel but negative signal in the CARD-FISH channel) were DOC-consuming heterotrophic prokaryotic cells. Note that only the oxic water column was sampled as we only assessed the aerobic methanotrophic and heterotrophic metabolisms in this study. Confining our sampling to the oxic portion of the water column avoided the inclusion of anaerobic prokaryotes in the DAPI-stained cells.

### Whole water column methanotrophy and heterotrophy

The whole water column methanotrophic C consumption in the six lakes was calculated by applying a model of methane oxidation rates to every meter (or 2 meters in L. Morency) of the water column using profiles performed in the summer of 2015 and 2016. The model used was developed on the same lakes and describes the kinetics of CH_4_ oxidation based on the local temperature and CH_4_ concentrations as well as the non-linear effect of O_2_ concentration following the equation of Thottathil et al.^17^:

1 $${{{{{\rm{ln}}}}}}\,{MOX}\,{rate}=\,	20.08+\left(0.79\times {{{{{\rm{ln}}}}}}\left[C{H}_{4}\right]\right)\\ 	 +\,\left(-5669.61\times \frac{1}{T}\right)\\ 	 +\,{{{{{\rm{ln}}}}}}({e}^{-0.01\times \left[{O}_{2}\right]}-{e}^{-\left(0.01+0.18\right)\times \left[{O}_{2}\right]})$$

where CH_4_ and O_2_ concentrations are in μmol L^−1^, temperature is in Kelvin, and methane oxidation rate (MOX rate) is in μmol L^−1^ d^−1^. Because the oxygen probe has an accuracy of 0.2 mg L^−1^ (YSI Rapid pulse sensor), O_2_ concentrations < 0.2 mg L^−1^ in profiles were considered anoxic. For that, we applied an offset correction to the oxygen profiles using a linear regression (${calibrated}\left[{O}_{2}\right]=-6.392+1.0228\times {measured}\left[{O}_{2}\right]$; O_2_ concentrations in μmol L^−1^). This resulted in a more conservative estimation of the role of aerobic methanotrophic at the lake level, since aerobic CH_4_ oxidation rates were only determined for the strictly oxic layer of the lakes.

For the upscaling of heterotrophy, we first determined the layers of thermally stratified water columns (epilimnion, metalimnion, and hypolimnion) using the monthly temperature profiles and the function meta.depths (minimum density slope = 0.1 kg m^−3^ m^−1^) from the R package rLakeAnalyzer (version 1.11.4.1)^73^. We used the default cutoff of 1 °C, which means that the profile was considered unstratified and no metalimnion depths were returned if the overall range in temperature was lower than 1 °C. Then, the total heterotrophic C consumption measured in incubations at different layers of the water column as described above were assumed to be constant within each stratified layer across the summer. For L. Triton and en Coeur, which were not stratified in most of the samplings, we used the depth of the bottom of the epilimnion in the few profiles showing thermal stratification to divide the water column into two layers and applied the correspondent measured heterotrophic metabolism rates to them.

Finally, the whole water column volume-weighted rates of both total methanotrophic and total heterotrophic C consumptions were determined for each profile by multiplying the rates by the volume of 1 or 2 m layer derived from lake-specific bathymetric maps (bathymetric maps were retrieved from the online repository at www.crelaurentides.org/dossiers/eau-lacs/atlasdeslacs). Then, we calculated the fraction of the total microbial C consumption (sum of methanotrophic and heterotrophic rates) consumed by methanotrophy and heterotrophy at the whole water column level for each profile of each lake.

### Data analysis

Data processing, calculations, and statistical analyses were performed using R (v4.0.4)^74^ in RStudio (v1.2.1335)^75^. Data processing was performed using the R package dplyr^76^, and plots were produced using the packages ggplot2, cowplot, gridExtra, and plotly^77–80^.

### Reporting Summary

Further information on research design is available in the Nature Research Reporting Summary linked to this article.

## Supplementary information

Supplementary Information

Reporting Summary

## Data Availability

The data generated in this study is available in the Zenodo public repository at 10.5281/zenodo.5737277^81^.

## Code availability

The code used for the analyses and figures reported in this manuscript can be found at https://github.com/paulacjr/Methanotrophy_in_lakes.
