# Supplementary material for: The role of methanotrophy in the microbial carbon metabolism of temperate lakes
Source: Nat Commun. 2022 Jan 10;13:43. doi: 10.1038/s41467-021-27718-2 (PMC8748455; doi:10.1038/s41467-021-27718-2)
Supplement: Supplementary file 1 — Supplementary Information [file 41467_2021_27718_MOESM1_ESM.pdf]

Supplementary information for:

The role of methanotrophy in the microbial carbon metabolism of temperate lakes

Paula C.J. Reis[1]*, Shoji D. Thottathil[2], Yves T. Prarie[1]

[1]Département des Sciences Biologiques, Groupe de Recherche Interuniversitaire en Limnologie, Université du Québec à Montréal, Montréal, QC, H2X 1Y4, Canada.

[2]Department of Environmental Science, SRM University AP, Amaravati, Andhra Pradesh 522 502, India.

*Corresponding author: paulacjr@gmail.com

Contents:

- Supplementary Table 1
- Supplementary Figure 1
- Supplementary Figure 2
- Supplementary Figure 3
- Supplementary Figure 4
- Supplementary Figure 5
- Supplementary Figure 6
- Supplementary Figure 7
- Supplementary Figure 8
- Supplementary Table 2
- Supplementary Table 3
- References

Supplementary Table 1. Area, maximum depth, and general characteristics of the surface waters of study lakes.

Lake	Area (km^2)	Max. depth (m)	TP (μg L^{-1})	TN (mg L^{-1})	Chl-a (μg L^{-1})	O_2 (mg L^{-1})*	CH_4 (μM)*	DOC (mg L^{-1})	cDOM (m^{-1})
Geai	0.0084	8.3	13.5	0.32	2.4	0.1-9.1	0.02-838	8.2	4.26
Cromwell	0.102	9.8	9.6	0.28	2.5	0.3-9.1	0.03-299	5.2	1.38
Croche	0.179	11.4	5.5	0.18	1.4	0.1-11.6	0.02-455	4.1	0.92
Morency	0.23	20.0	5.0	0.23	1.4	0.3-18.8	0.01-221	3.4	0.69
en Coeur	0.44	8.3	6.9	0.25	1.6	0.6-9.5	0.07-2.2	4.2	0.46
Triton	0.017	4.3	10.3	0.29	2.2	5.5-9.7	0.3-1.7	4.6	1.04

TP: total phosphorus; TN: total nitrogen; Chl-a: chlorophyll-a; DOC: dissolved organic carbon; cDOM: colored dissolved organic matter (absorbance at 440 nm)[1]. (*) Dissolved oxygen (O_2) and methane (CH_4) values shown are the minimum and maximum measured along the water column across the summer season.

Supplementary Figure 1. Change in the instantaneous methane oxidation rate – which was determined by multiplying the first-order rate constant (*k*) by the methane concentration at each time point of the incubations – in each lake and water column layer. The instantaneous rates reduce over the course of incubations due to the reduction in methane concentration. Texts in each plot indicate the total duration of incubations. Note log scale of y axis.

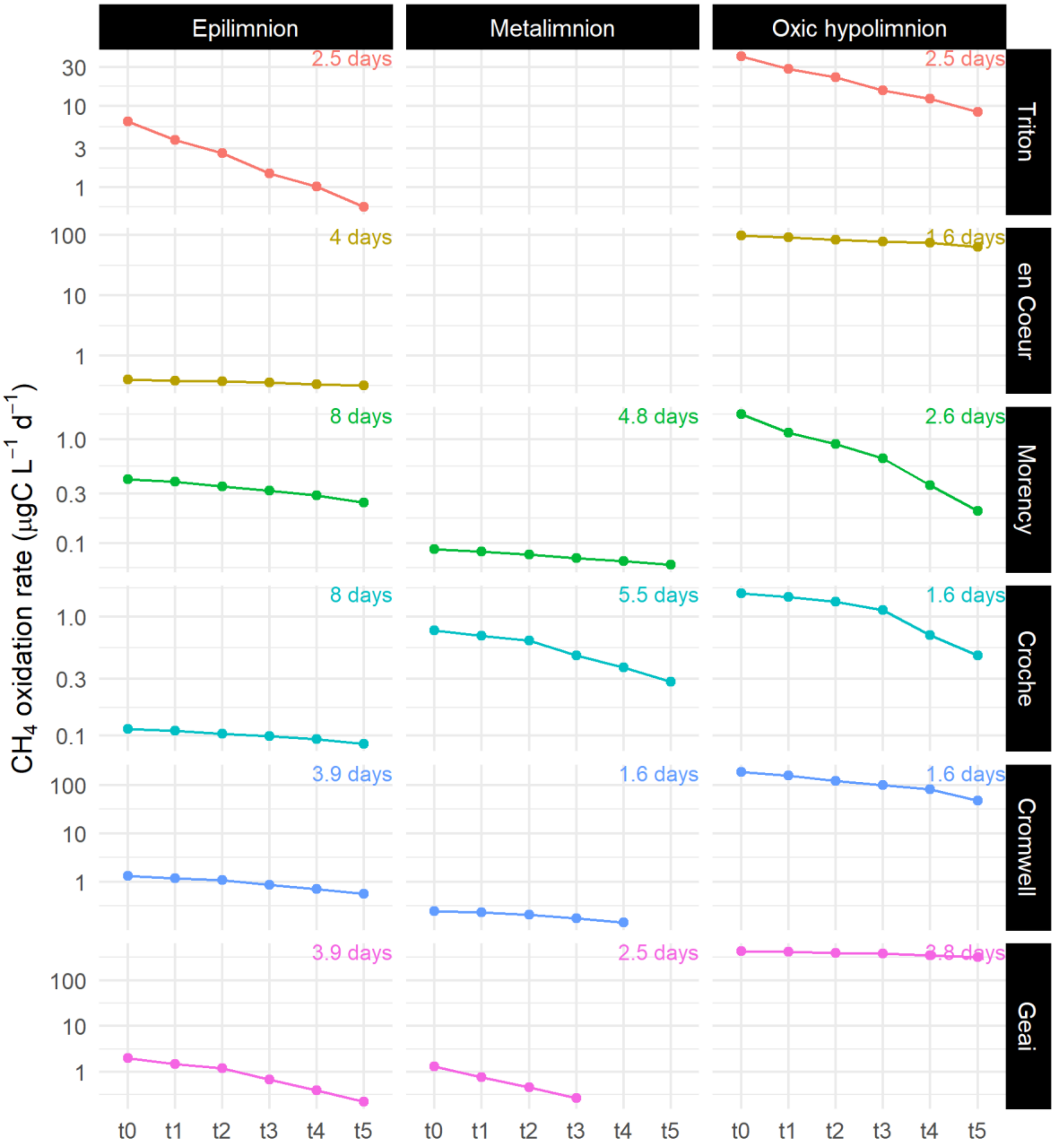

Supplementary Figure 2. Heterotrophic metabolism measurements at each sampled depth in the six studied lakes. The heterotrophic metabolism was determined as the sum of microbial production (incorporation of ^{3}H-leucine) and microbial aerobic respiration (oxygen consumption in 1.2 µm-filtered waters). See Methods in the main text for details.

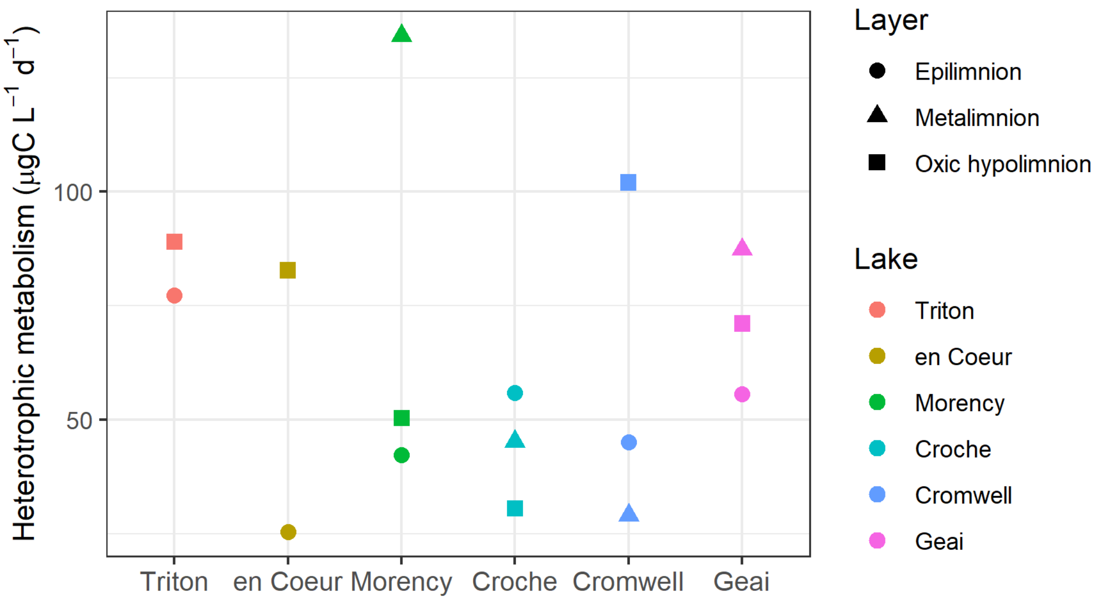

Supplementary Figure 3. Change in carbon (C) consumption by methane-oxidizing bacteria (MOB) per unit biomass (specific activity) along the gradient in CH_4 concentration across incubations. Plotted trend line is a loess curve and shaded area around the line indicates 95% confidence interval. Color of points shows the O_2 concentration. Note log scale of y and x axes.

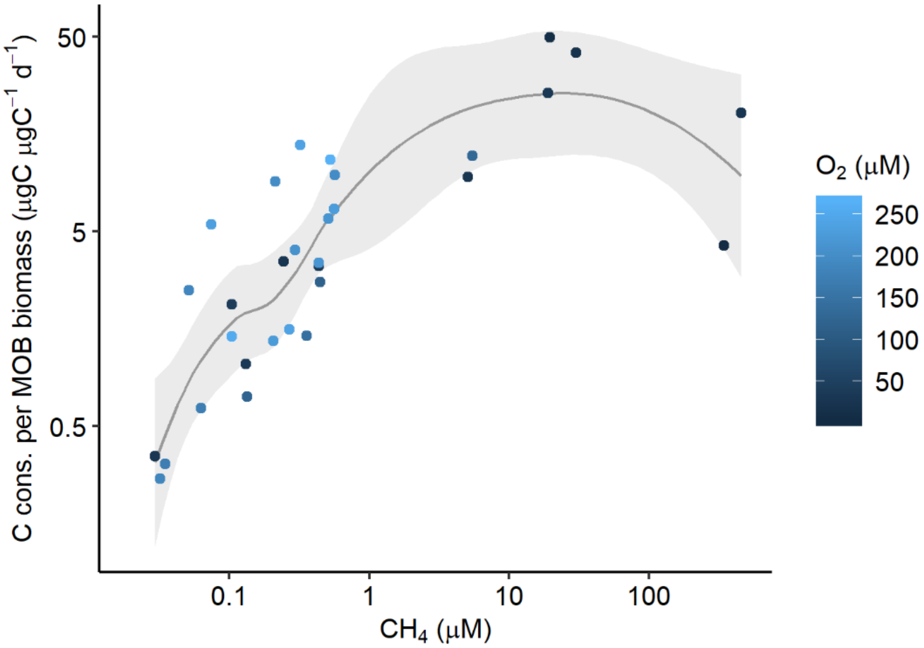

Supplementary Figure 4. Vertical profiles of methane (CH_4), oxygen (O_2), and temperature taken at the early, mid, and late summer in 2015 in each studied lake (profiles were sampled monthly from May to November, but only three profiles are shown for clarity). Grey shaded areas indicate the layer of CH_4:O_2 molar ratio > 0.6 measured at the early, mid, and late stratification in that year. Note that the extent of the layer of CH_4:O_2 molar ratio > 0.6 increases from the early (darkest shaded area) to the late stratification (lighter shaded area).

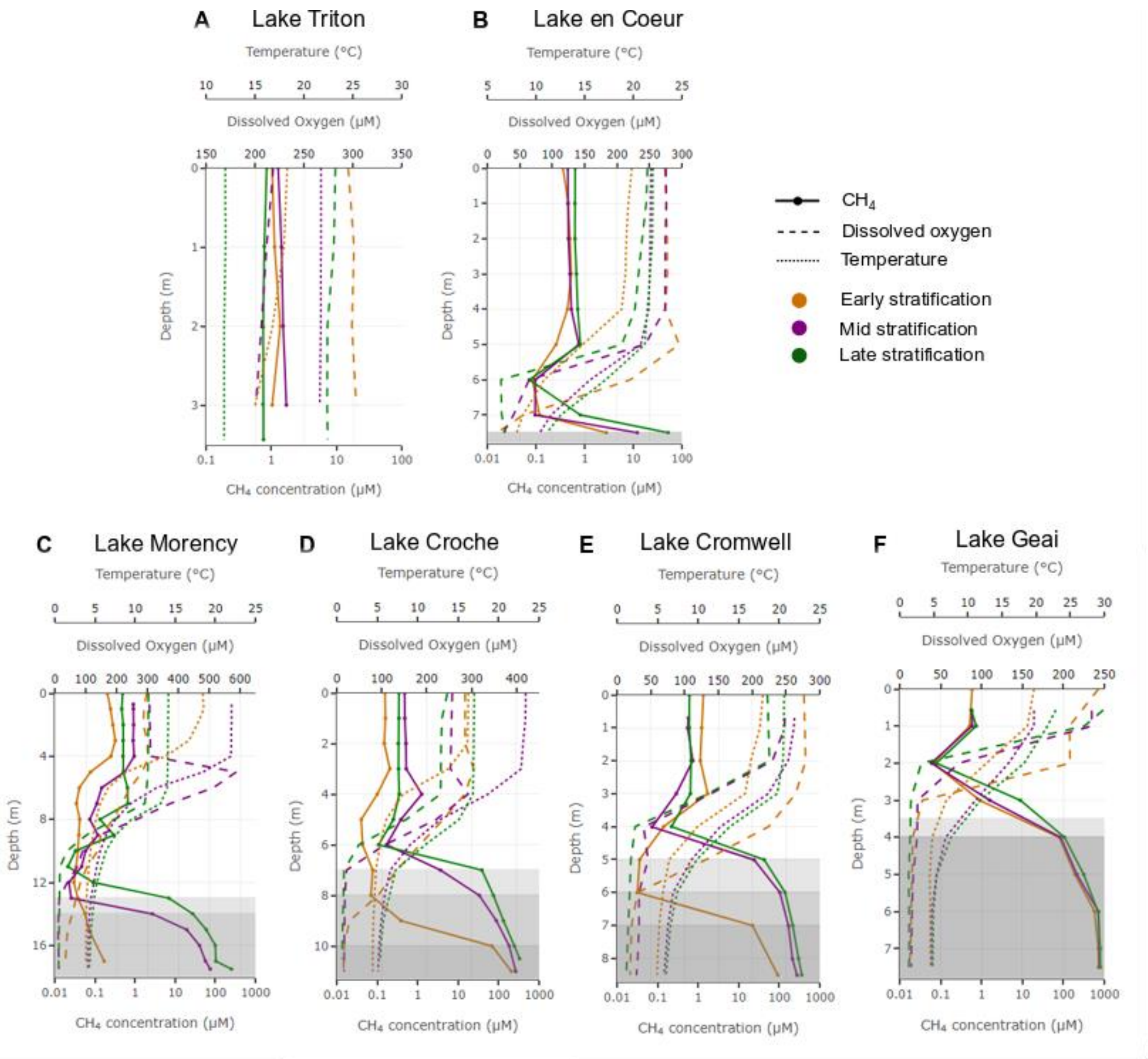

Supplementary Figure 5. Relationships between dissolved organic carbon, water color, and light penetration in the studied lakes. A) Dissolved organic carbon (DOC) concentration and colored dissolved organic matter (cDOM) ($p < 0.001$, F-test (F-statistic: 1694, d.f. = 48)). B) cDOM and the attenuation coefficient of photosynthetically active radiation (K_d PAR) ($p < 0.001$, F-test (F-statistic: 420, d.f. = 48)). Lines indicate linear relationship including all studied lakes (well-stratified and not stratified). cDOM was determined as the light absorbance at 440 nm[1].

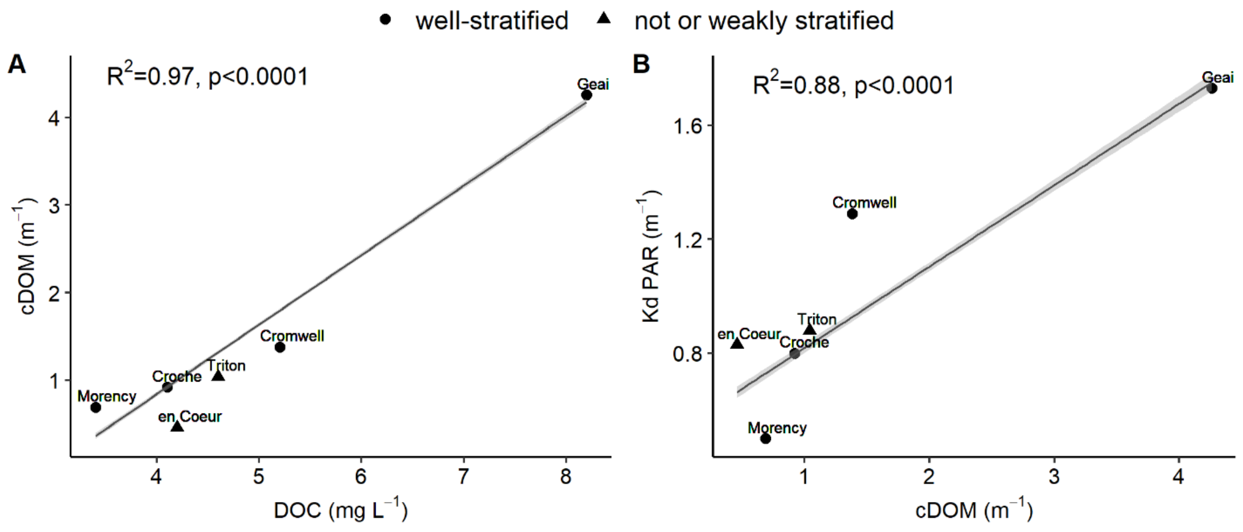

Supplementary Figure 6. Cell abundance of methane-oxidizing bacteria (MOB) and other prokaryotes at the start ('Initial) and end ('Final') of incubations for the determination of methane oxidation rates (unfiltered) in the six studied lakes. MOB and other prokaryotes were detected and enumerated microscopically by catalyzed reporter deposition-fluorescence in situ hybridization (CARD-FISH) and 4',6-diamidino-2-phenylindole (DAPI) staining, respectively. Boxplots represent median, first and third quartiles (hinges), and 1.5 x interquartile range (whiskers) of three (L. Croche, en Coeur, Morency; n = 3) or two (L. Cromwell, Geai, Triton; n = 2) independent replicates (bottles). The lakes not showing clear stratification (L. Triton and L. en Coeur) were not sampled in the metalimnion. No consistent changes were observed between the average initial and final abundance of MOB and DAPI cells.

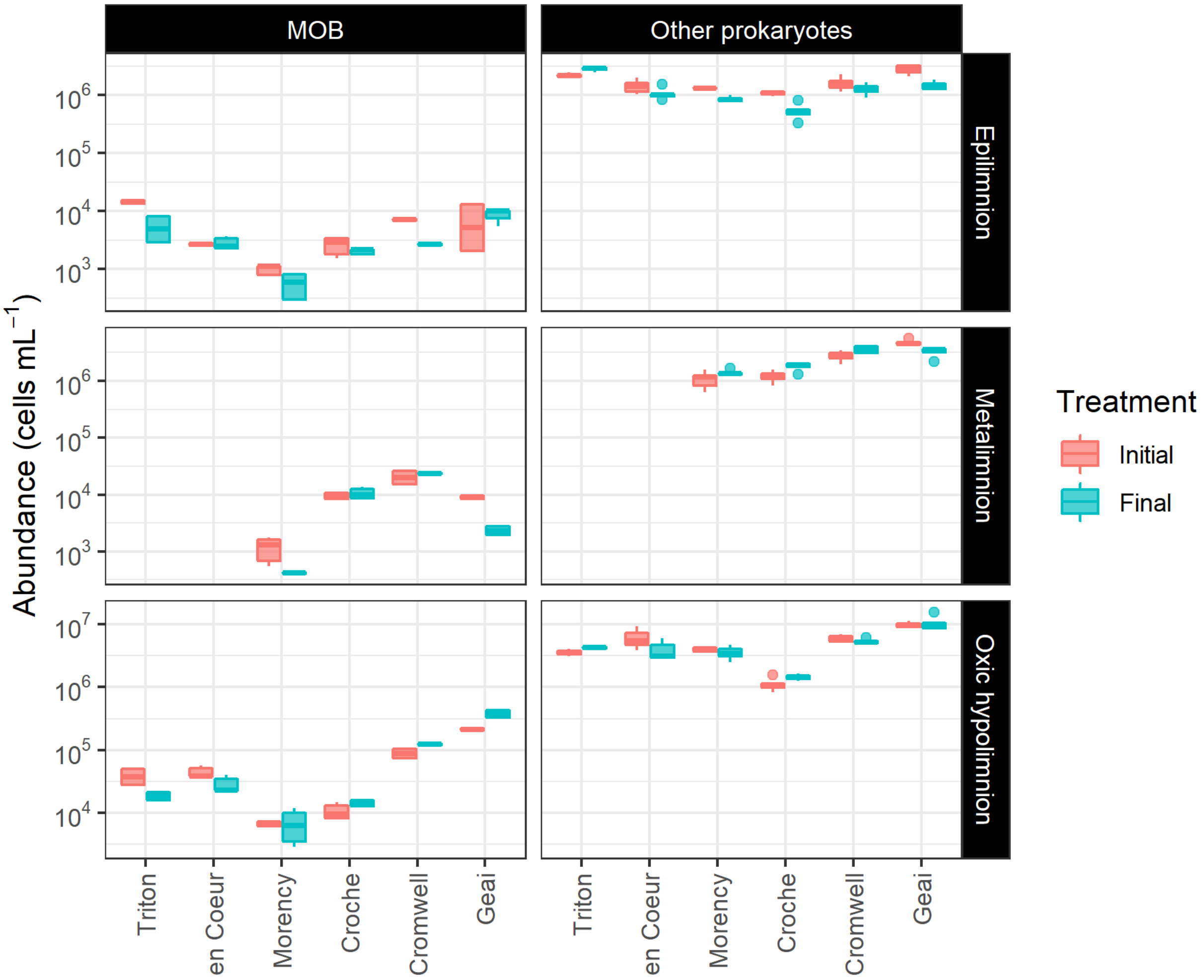

Supplementary Figure 7. Dissolved oxygen and methane concentrations at sampling (lake) and at the start of incubations (t0 incubation) showing that in the vast majority of the cases the in situ gas concentrations did not change substantially during sampling, processing time, and handling of water samples.

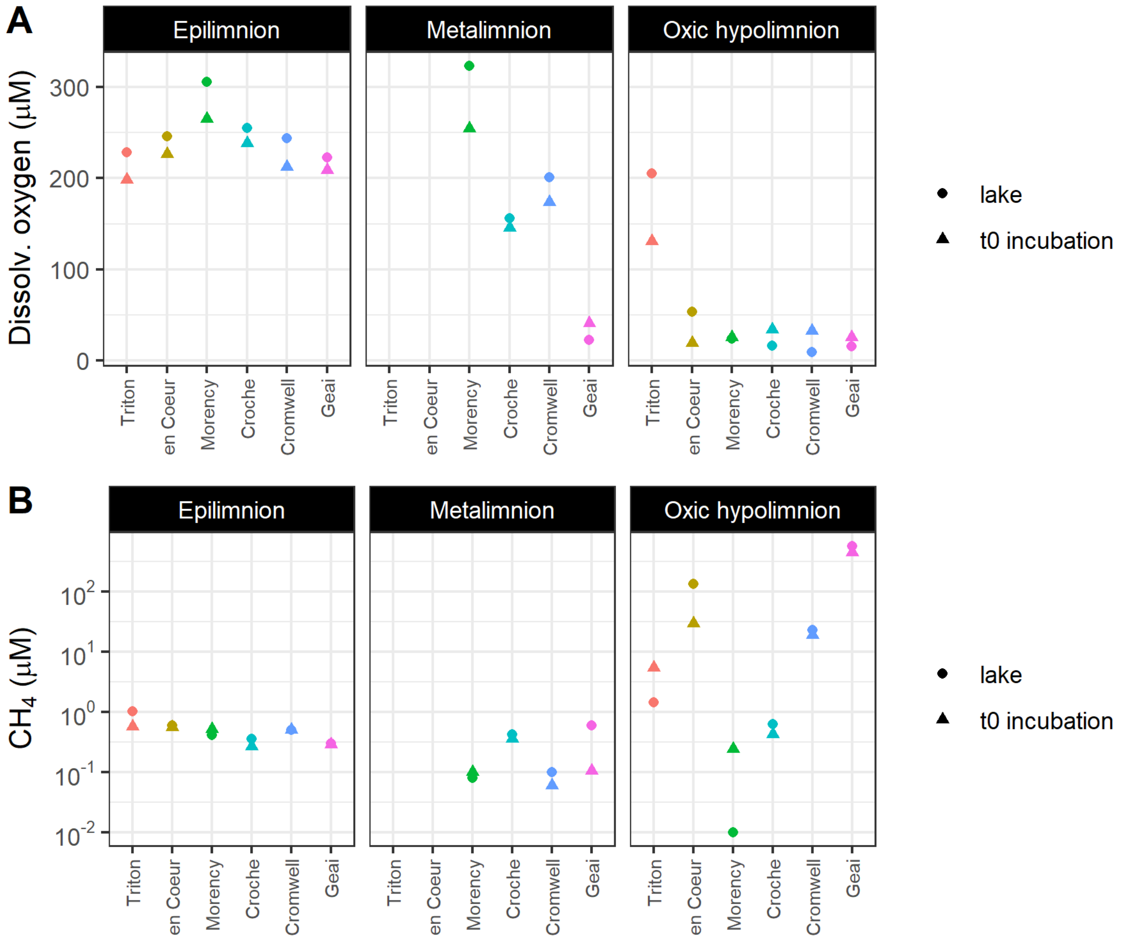

Supplementary Figure 8. Change in total biomass of methane-oxidizing bacteria (MOB) and other prokaryotes due to filtration at 1.2 µm pore size of water from the different sampled lakes and depths at the start of incubations. MOB and other prokaryotes were detected and enumerated microscopically by catalyzed reporter deposition-fluorescence in situ hybridization (CARD-FISH) and 4’,6-diamidino-2-phenylindole (DAPI) staining, respectively. Total biomass was calculated from cell size as explained in the Methods section. Boxplots represent median, first and third quartiles (hinges), and 1.5 x interquartile range (whiskers) of three (L. Croche, en Coeur, Morency; n = 3) or two (L. Cromwell, Geai, Triton; n = 2) independent replicates (bottles). Note that the lakes not showing well-defined stratification layers (L. Triton and L. en Coeur) were not sampled in the metalimnion. Filtered biomass for the metalimnion and oxic hypolimnion of L. Geai could not be determined due to technical issues.

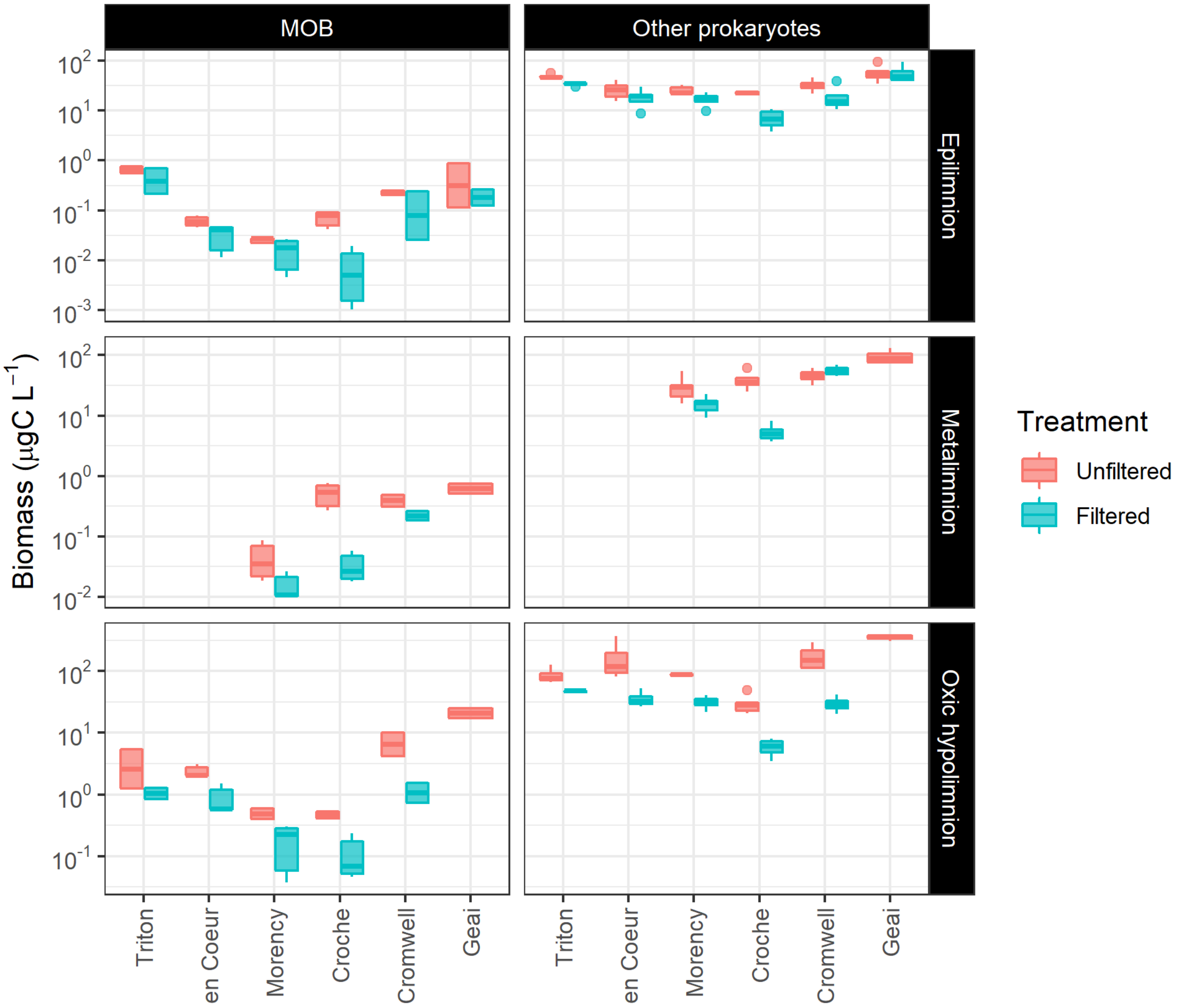

Supplementary Table 2. Probes used for the detection of methane-oxidizing bacteria (MOB) in lake water samples through catalyzed reporter deposition-fluorescence in situ hybridization (CARD-FISH)[2] and their coverage of common freshwater MOB taxa. Probe coverage was verified on TestProbe 3.0 on Silva's SSU r138.1 on June 8th, 2021.

**Probe name**	**Probe sequence**	**Target group**<br>**[Main genera (probe coverage)]**
Malpha450	5'-ATC CAG GTA CCG TCA TTA TC-3'	Alphaproteobacteria MOB<br>*[Methylocystis* (94%), Methylosinus (94%)]*
Mgamma84	5'-CCA CTC GTC AGC GCC CGA-3'	Gammaproteobacteria MOB<br>*[Methylobacter* (83%), Methylococcus (94%), Methylomonas (61%), Methylosarcina (100%), Crenothrix* (53%)]*
Mgamma705	5'-CTG GTG TTC CTT CAG ATC-3'	Gammaproteobacterial MOB<br>*[Methylomonas (100%), Methylosphaera (100%), Methylovulum (63%), Methylobacter* (42%), Methylomicrobium (29%), Crenothrix* (15%)]*

* Methanotrophic genera detected in 16S rDNA amplicon sequencing samples from two of the studied lakes (L. Croche and Cromwell). Water samples were collected from the epilimnion and hypolimnion from May to October 2018 and were processed and analyzed as described in Reis et al.[3]

Supplementary Table 3. Maximum relative abundance of non-target taxa (non-MOB taxa covered by the CARD-FISH probes used in this study) in 16S rDNA amplicon sequencing samples from two of the studied lakes. The table also shows the coverage of the non-target taxa by the primers used in the 16S rDNA sequencing. Note that non-target taxa are absent or present in very low abundances in the 16S rDNA sequencing data (first column), while the primers used would have detected them if present (second and third column). Probes and primers' coverage checks were performed on TestProbe 3.0 on Silva's SSU r138.1 on June 8th 2021 and September 17th 2021, respectively.

**Probe**	**Non-target taxa**	**Max. rel. abundance (% of 16S rDNA seqs.)**	**515F primer coverage (% of seqs. in Silva)**	**806R primer coverage (% of seqs. in Silva)**
**Malpha450**	*Parvibaculales*	0	96.7	92.6
	alphaI cluster	0	94	96
	Bauldia	0	100	94.2
	Roseiarcus	0	91.7	97.9
	Youngimonas	0	100	100
**Mgamma84**	*Macromonas*	0	75	75
	Ectothiorhodospirales	0	97	90.7
	Halothiobacillales	0	93.9	92.7
	Salinispira	0.44	90.9	90.9
	Tenderiales	0	90.9	87.9
	Plasticicumulans	0	100	100
	Hydrogenophaga	0	95.7	95.3
	Beggiatoaceae	1.4	96.4	97.8
**Mgamma705**	*Pseudomonadales*	1.3	93.2	91.2
	Planctomicrobium	0	92.2	93.5
	Thiotrichales	0	97.3	94.7

References

1. Cuthbert, I. D. & del Giorgio, P. Toward a standard method of measuring color in freshwater. *Limnol. Oceanogr.* **37**, 1319–1326 (1992).

2. Eller, G., Stubner, S. & Frenzel, P. Group-specific 16S rRNA targeted probes for the detection of type I and type II methanotrophs by fluorescence in situ hybridisation. *FEMS Microbiol. Lett.* **198**, 91–97 (2001).

3. Reis, P. C. J., Ruiz-González, C., Soued, C., Crevecoeur, S. & Prairie, Y. T. Rapid shifts in methanotrophic bacterial communities mitigate methane emissions from a tropical hydropower reservoir and its downstream river. *Sci. Total Environ.* **748**, (2020).
