# Supplementary material for: The role of methanotrophy in the microbial carbon metabolism of temperate lakes
Source: Nat Commun. 2022 Jan 10;13:43. doi: 10.1038/s41467-021-27718-2 (PMC8748455; doi:10.1038/s41467-021-27718-2)
Supplement: Supplementary file 2 — Reporting Summary [file 41467_2021_27718_MOESM2_ESM.pdf]

nature portfolio

Corresponding author(s): Paula Reis

Last updated by author(s): Nov 29, 2021

Reporting Summary

Nature Portfolio wishes to improve the reproducibility of the work that we publish. This form provides structure for consistency and transparency in reporting. For further information on Nature Portfolio policies, see our Editorial Policies and the Editorial Policy Checklist.

Statistics

For all statistical analyses, confirm that the following items are present in the figure legend, table legend, main text, or Methods section.

n/a	Confirmed	
☐	☒	The exact sample size (n) for each experimental group/condition, given as a discrete number and unit of measurement
☐	☒	A statement on whether measurements were taken from distinct samples or whether the same sample was measured repeatedly
☐	☒	The statistical test(s) used AND whether they are one- or two-sided<br>*Only common tests should be described solely by name; describe more complex techniques in the Methods section.*
☒	☐	A description of all covariates tested
☐	☒	A description of any assumptions or corrections, such as tests of normality and adjustment for multiple comparisons
☐	☒	A full description of the statistical parameters including central tendency (e.g. means) or other basic estimates (e.g. regression coefficient) AND variation (e.g. standard deviation) or associated estimates of uncertainty (e.g. confidence intervals)
☐	☒	For null hypothesis testing, the test statistic (e.g. F, t, r) with confidence intervals, effect sizes, degrees of freedom and P value noted<br>*Give P values as exact values whenever suitable.*
☒	☐	For Bayesian analysis, information on the choice of priors and Markov chain Monte Carlo settings
☒	☐	For hierarchical and complex designs, identification of the appropriate level for tests and full reporting of outcomes
☒	☐	Estimates of effect sizes (e.g. Cohen's d, Pearson's r), indicating how they were calculated

Our web collection on statistics for biologists contains articles on many of the points above.

Software and code

Policy information about availability of computer code

Data collection	The ACME tool3 software (2014) was used to count cells and to measure their sizes.
Data analysis	Data analysis and visualization was performed using R version 4.0.4 in RStudio (v1.2.1335). R packages used: dplyr (v1.0.5), rLakeAnalyzer (v1.11.4.1), ggplot2 (v3.3.3), cowplot (v1.1.1), gridExtra (v2.3), and plotly (v4.9.4.1).

For manuscripts utilizing custom algorithms or software that are central to the research but not yet described in published literature, software must be made available to editors and reviewers. We strongly encourage code deposition in a community repository (e.g. GitHub). See the Nature Portfolio guidelines for submitting code & software for further information.

Data

Policy information about availability of data

All manuscripts must include a data availability statement. This statement should provide the following information, where applicable:

- Accession codes, unique identifiers, or web links for publicly available datasets
- A description of any restrictions on data availability
- For clinical datasets or third party data, please ensure that the statement adheres to our policy

The data generated in this study have been deposited in the Zenodo database under accession code ZZ [add hyperlink here].

Field-specific reporting

Please select the one below that is the best fit for your research. If you are not sure, read the appropriate sections before making your selection.

☐ Life sciences ☐ Behavioural & social sciences ☒ Ecological, evolutionary & environmental sciences

For a reference copy of the document with all sections, see nature.com/documents/nr-reporting-summary-flat.pdf

Ecological, evolutionary & environmental sciences study design

All studies must disclose on these points even when the disclosure is negative.

Study description

We performed laboratory incubations, microscopic cell counts and area measurements, and lake water column vertical profiles. Incubations were performed to measure methanotrophy and heterotrophy metabolic rates in lake water. Methanotrophic rates were determined by the reduction in methane concentration over time measured in duplicate bottles at each time point (incubation was interrupted in two bottles at each time point). Heterotrophic rates were determined by 3H-leucine incorporation (production) and by oxygen consumption over time in incubations (respiration) (the same duplicate flaks equipped with O2 optodes were measured at each time point). Methanotrophic and total bacterial cells were enumerated and had their area determined microscopically at the start and end of each incubation (CARD-FISH and DAPI staining were used). Multiple profiles of water temperature, oxygen and methane concentrations were performed in each of the six studied lakes at least 4 and up to 13 times over the summer stratification period of 2015 and 2016. We applied a model of methane oxidation rates developed in a parallel study to the profiles and used bathymetric maps of each lake to obtain volume-weighted rates and upscale the relative importance of methanotrophy at the lake scale.

Research sample

Samples were comprised of lake water and bacterioplankton from 2 or 3 depths of six temperate lakes located in the Laurentians region of Quebec, Canada. The lakes were chosen to cover a large range in dissolved organic carbon concentration, methane concentration and morphometry. Sampling depths for incubations were determined based on temperature and dissolved oxygen profiles in order to cover the stratified layers and a large range in oxygen and methane concentrations. In lakes showing three well-defined layers, we took samples from the epilimnion, metalimnion and oxic hypolimnion. In the other lakes, we took samples from the subsurface (corresponding to epilimnion) and oxic bottom (corresponding to oxic hypolimnion). The samples aimed to represent each layer of the water column of the sampled lakes during the summer period.

Sampling strategy

We sampled six temperate lakes located in the Laurentians region of Québec, Canada during the summer in 2015 and 2016. These lakes were chosen for differing largely in size, maximum depth, DOC concentration, and other characteristics. In 2015, water column profiling of temperature, O2, and CH4 concentrations were performed in the six studied lakes from May to November. Additional profiles were taken in 2016 between June and September in L. Croche and Geai. Temperature and O2 measurements were made at each meter using a YSI probe (Yellow Springs Instruments, USA; O2 detection limit: 0.2 mg L-1) and the concentration of CH4 was determined using the headspace technique followed by gas chromatography (GC; 8A/GC-2014, Shimadzu, Japan; CH4 detection limit: 0.1 ppm). Vertical profiles of photosynthetically active radiation (PAR) were performed once in each lake in 2015 using an underwater light sensor (LI-192, LI-COR Biosciences, USA). Incubations for the determination of methanotrophy and heterotrophy C consumptions were performed during the summer in 2016. For this, water was collected with a peristaltic pump from the epilimnion, metalimnion, and oxic hypolimnion in the lakes showing three well-defined layers (Morency, Croche, Cromwell, and Geai) and from the subsurface and bottom in lakes Triton and en Coeur, covering a large gradient in CH4 and O2 concentrations (0.02–455 μM and 3–265 μM, respectively). The water was pumped into acid-washed collapsible bags until overflow and were kept cold and in the dark for 2 to 4 h until arrival at the laboratory.

In the laboratory, ten 500-mL flasks equipped with O2 optodes (Fibox 3, PreSens, Germany) per sampled depth were filled with lake water allowing to overflow and sealed with silicone stoppers without headspace. Flasks were incubated at in situ temperature (± 2°C) in dark circulation water baths for at least 1.6 and up to 8 days, and duplicate flasks were sampled three to five times during each incubation for the determination of CH4 concentration. Low standard deviations from mean CH4 concentrations (<10%) and isotopic signatures (<5%) between replicates confirmed that the experimental setup was adequate. Microscopic verification showed that MOB abundance did not change substantially during most incubations despite the duration of incubations. CH4 concentration was measured by the headspace equilibration technique in 60 mL gas tight syringes with ultra-high purity zero air (Praxair Canada Inc., Canada) (1:1 water sample and zero air ratio). Syringes were vigorously shaken by 2 min and then the headspace was transferred to pre-evacuated vials (Labco Ltd. UK), where samples were kept until analysis. CH4 partial pressure in the headspace gas was measured through cavity ring down spectrometry (CRDS – Picarro G2201-i, Picarro Inc, USA) or gas chromatography in the case of samples exceeding 200 µatm of CH4 in the headspace gas because of possible interference of laser paths in CRDS at high CH4 concentrations. The dissolved CH4 concentration in the water was then calculated by multiplying the CH4 partial pressure by a temperature dependent Henry's law constant. CH4 oxidation rates were determined by multiplying the slope of the first order CH4 concentration decay curve (k) by the observed CH4 concentration at each time point of the experiment. This approach allowed that the rates were attributed to the current MOB community, which was quantified at the initial and final time point of incubations. In addition, the decay curves of CH4 concentration over time were near perfect log-linear in incubations (R2 > 0.92), implying that different incubation duration would not have yielded different rate constants (k). Moreover, the measured CH4 oxidation rates represent actual rather than potential rates since the incubations were performed at in situ CH4 and O2 concentrations with minimal changes in concentration between the sampling and the start of incubations and in the presence of potential grazers (unfiltered waters). CH4 concentration and carbon stable isotopic signature were significantly (p < 0.0001) and strongly (R2 > 0.86) linearly correlated across incubations, which indicated that decreases in CH4 concentration were due to microbial CH4 consumption and that CH4 production was very unlikely or minimal in the incubations.

Data collection

PCJR and SDT collected and curated the data. Lake profiles and samples were collected in the field at the deepest point of the studied lakes. Incubation data were collected in the laboratory. Incubations for methanotrophic rate and heterotrophic respiration rate were measured at least 3 and up to 5 times and lasted between 1.6 to 8 days. Heterotrophic production rates were measured by the

	incorporation of 3H-leucine in incubations of 1 h following Smith and Azam (1992).
Timing and spatial scale	Water column vertical profiles of temperature, O2, and CH4 concentrations were performed at the deepest point of each lake monthly from May to November in 2015; additional profiles were taken during the summer 2016. Measurements were performed every meter or every 2 meter for the deepest lake. We used a monthly frequency in 2015 to capture the progress of the thermal stratification and the changes in the O2 and CH4 profiles along the season, which could have profound impacts on the rate and spatial extent of methanotrophy in the water column. Samples for incubations were collected from the deepest point of the lake during summer 2016 in each stratified layer in the lakes showing well-defined stratification layers and in two depths (subsurface and bottom) in the other lakes. Incubations for methanotrophic and heterotrophic metabolisms were started in the lab within 2-4h after sampling. Incubations for methanotrophy were held for at least 1.6 and up to 8 days, depending on the pace of CH4 oxidation in the flasks. To determine that, at each time point CH4 concentration and stable isotopic signature were measured right after sampling flaks for verification of significant CH4 consumption. The time period between time points was then adjusted based on the change in CH4 concentration since the previous time point and incubations were sampled at least three and up to five times. This allowed the detection of significant reduction in CH4 concentration and stable isotopic enrichment in every incubation series and avoided that we did not detect CH4 oxidation due to too short incubations. Water samples for microscopy were collected at the start and end of incubations from each flask and were fixed overnight. Filters were frozen until the microscopic analyses, which were performed in Feb-March 2017.
Data exclusions	No data were excluded from the analyses.
Reproducibility	Every experiment was performed using duplicate (methanotrophic rate and heterotrophic respiration) or triplicate (heterotrophic production) flasks. All replicates were successful.
Randomization	The samples were grouped depending on the lake and depth of origin. Randomization was not relevant to this study.
Blinding	Blinding was not relevant to this study as there were no risks of bias related to control versus treatment groups in experiments.

Did the study involve field work? [x] Yes [ ] No

Field work, collection and transport

Field conditions	This study was performed during the ice-free period in the Laurentians region Quebec, Canada (May-November). The average air temperature during sampling was 18 degrees C. Sampling was not performed during raining days.
Location	The lakes sampled in this study are located in or in the surroundings of the Station de biologie des Laurentides of the Univeriste de Montreal (45°59'17.8"N 74°00'20.9"W). The mean elevation in the area is 350 m. Two to three water depths were sampled in each lake depending on the temperature and oxygen vertical profiles.
Access & import/export	The lakes were accessible by road and/or walk. No import/export permits of samples were needed.
Disturbance	To avoid potential dispersion of species, the boat was rinsed prior to entering a lake when the same boat had to be used for sampling in different lakes. Paddles were used instead of a motor in the shallower and smaller lakes to avoid sediment resuspension and shore deterioration. Any disposable material used in this study was recycled when possible, and all chemical dejects produced (e.g. during preparation of samples and slides for microscopic analysis) were disposed following the Universite du Quebec a Montreal 's waste disposal protocols.

Reporting for specific materials, systems and methods

We require information from authors about some types of materials, experimental systems and methods used in many studies. Here, indicate whether each material, system or method listed is relevant to your study. If you are not sure if a list item applies to your research, read the appropriate section before selecting a response.

Materials & experimental systems

n/a	Involved in the study
[x]	[ ] Antibodies
[x]	[ ] Eukaryotic cell lines
[x]	[ ] Palaeontology and archaeology
[x]	[ ] Animals and other organisms
[x]	[ ] Human research participants
[x]	[ ] Clinical data
[x]	[ ] Dual use research of concern

Methods

n/a	Involved in the study
[x]	[ ] ChIP-seq
[x]	[ ] Flow cytometry
[x]	[ ] MRI-based neuroimaging
